# Lamin B1 loss in human cardiomyocytes is associated with nuclear shape deformation in dilated cardiomyopathy

**DOI:** 10.1016/j.jmccpl.2026.100865

**Published:** 2026-08-17

**Authors:** Leandro N. Ventimiglia, Aleksej Zelezniak, Cristobal G. dos Remedios, Sean Lal, Elisabeth Ehler, Gian F. De Nicola

**Affiliations:** a The Randall Centre for Cell & Molecular Biophysics, New Hunt's House, Guy's Campus, King's College London, SE1 1UL, UK; b Sydney Heart Bank, Discipline of Anatomy & Histology, University of Sydney, Sydney, Australia; c British Heart Foundation Centre of Research Excellence, School of Cardiovascular and Metabolic Medicine and Sciences, King's College London, SE1 1UL, UK

**Keywords:** Nuclear lamina, Nuclear shape, Lamin B1, Cardiomyocyte, Dilated cardiomyopathy, Morphological phenotyping

## Abstract

Dilated cardiomyopathy (DCM) is a progressive heart muscle disease characterized by the enlargement of the heart's left ventricle. The aetiology of the disease can be genetic and/or environmental. Mutations in LMNA, the gene encoding for the nuclear lamina protein lamin A/C, are associated with approximately 6% of DCM, however, the role of the functionally related lamin B1 remains unknown. Lamin B1 provides structural integrity to the nucleus and regulates gene expression by restricting the accessibility and transcription of lamin B1 tethered genes. Our study focuses on the lamin B1 content and nuclear shape in human DCM. We first compare the levels of lamin B1 expression at the protein level in non-failing versus DCM diagnosed human hearts and then use an unbiased immunofluorescence and morphological phenotyping approach of cardiomyocyte nuclei to classify subpopulations of cells. This revealed that hearts diagnosed with DCM show a higher prevalence of nuclei within cardiomyocytes that were deformed and had a loss of lamin B1 signal. Using our unbiased analysis of the cardiomyocyte nuclei, we can separate non-failing from DCM donors.

In conclusion, our study shows that lamin B1 loss in human cardiomyocytes is associated with nuclear shape deformation in dilated cardiomyopathy.

## Highlights

• Lamin B1 loss is observed in human DCM cardiomyocytes.
• Nuclear shape is deformed in human DCM cardiomyocytes.
• Nuclear phenotyping analysis separates DCM from non-failing human hearts.

## Graphical abstract

**Figure:**
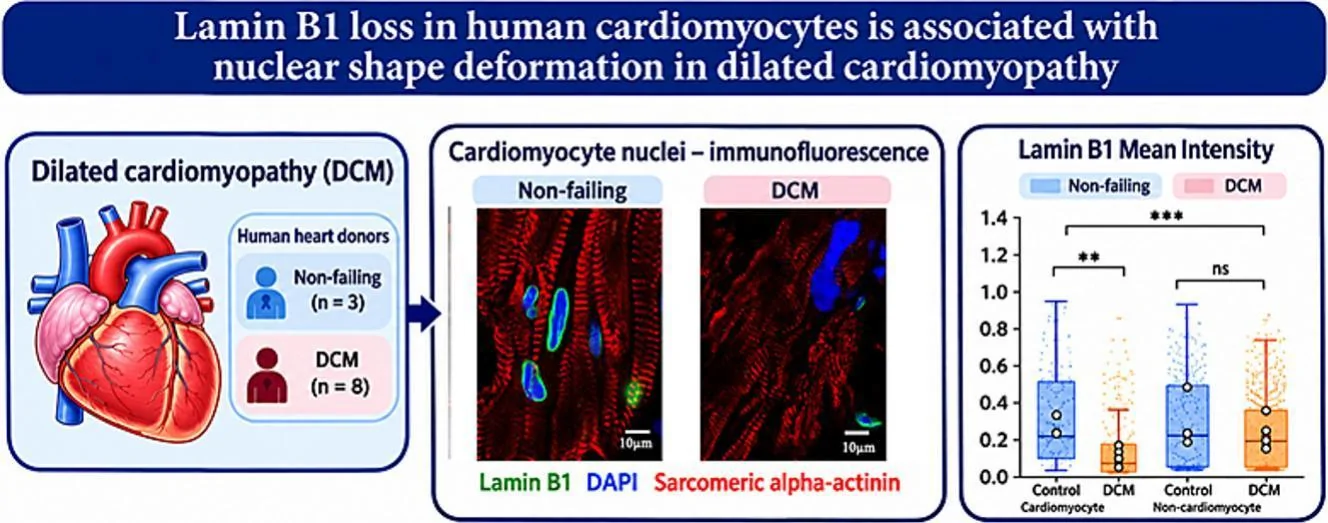

## 1 Introduction

Dilated cardiomyopathy (DCM) is a progressive heart muscle disease characterized by the enlargement of the heart's left ventricle. This dilation impairs the heart's ability to pump blood efficiently, frequently resulting in heart failure. The aetiology of the disease can be both genetic and environmental, however in more than 50% of the cases the cause remains unknown [1].

Mutations in LMNA are associated with approximately 6% of the cases of dilated cardiomyopathy (DCM) [2], [3], however the role of the functionally related lamin B1 protein is not well defined.

Lamin A/C is encoded by gene LMNA, whereas lamin B1 is encoded by the gene LMNB1, both proteins are part of the nuclear lamina. The nuclear lamina is a network of intermediate filaments that lies beneath the inner nuclear membrane, it plays a critical role in integrating cytoskeletal dynamics within the cell and in regulating the shape and structural integrity of the nuclear envelope as well as gene expression through chromatin interactions [4], [5].

Lamin B1 is an extremely long-lived protein whose concentration is tightly controlled within the cell [6]. Lamin B1 is constitutively expressed in somatic cells in the heart.

Disruption of lamin B1 integrity is reported in the context of apoptosis, where it is mediated through caspase 3 and caspase 6 [7], [8], and during cell division, where lamin B1 phosphorylation leads to its disassembly [9]. Autophagy is also a mechanism described for the degradation of lamin B1, where it has been observed in fibroblasts in the context of oncogene-induced senescence [10].

Still, very little is known about lamin B1 homeostasis in post-mitotic cells. Recently, Casterton et al. identified a novel mechanism of cell degeneration and death in neurons, named karyoptosis, that stems from proteotoxic stress caused by protein aggregates and/or lysosomal autophagic clearance impairment. The authors show that the main driver of karyoptosis is lamin B1, where loss through MAPK p38 alpha activation, leads to nuclear deformation and eventually cell death. Unbiased immunofluorescence staining and morphological phenotyping of neuronal nuclei in human brains diagnosed with fronto-temporal dementia and Alzheimer's disease identified populations of cells, which display features consistent with karyoptosis (i.e. lamin B1 loss and nuclear shape deformation) [11].

Here we present a pilot study using three non-failing and eight DCM heart donors; we first compare the expression levels of lamin B1 in non-failing versus DCM-diagnosed human hearts by immunoblotting whole heart extracts. We then use immunofluorescence quantification of lamin B1 and morphological phenotyping of cardiomyocyte nuclei to determine whether we can use those features to identify clusters within cardiomyocyte nuclei populations. This revealed that hearts diagnosed with DCM show a higher prevalence of cardiomyocytes belonging to a cluster defined by deformed nuclei and loss of lamin B1. Finally, we can distinguish between non-failing donors and end-stage DCM samples using unbiased analysis of their cardiomyocyte nuclei.

## 2 Results

### 2.1 Lamin B1 quantification in cardiomyocytes and whole heart tissue homogenate

**Fig. 1:**
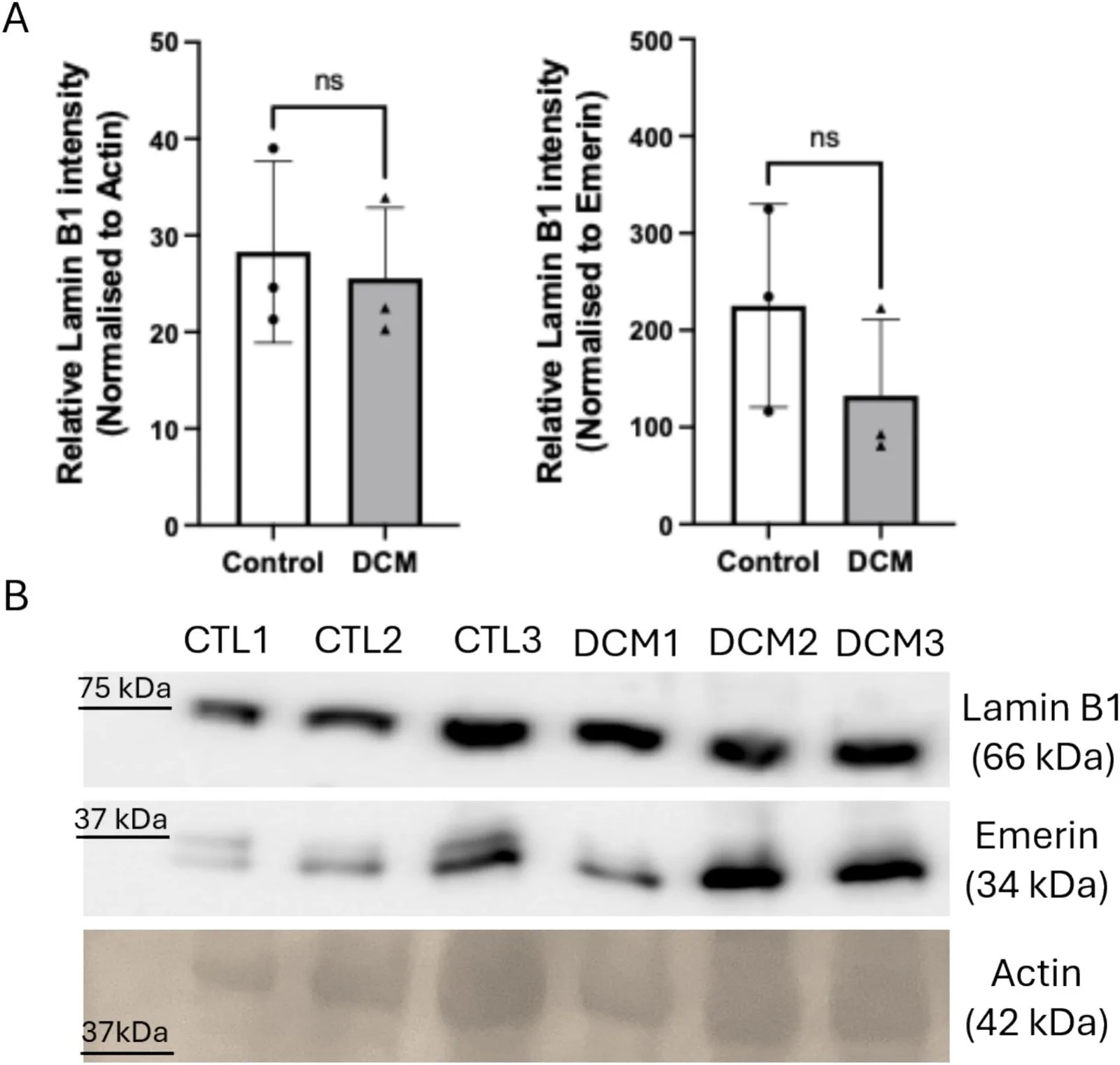
Lamin B1 quantification in human heart homogenates. Human myocardium tissue homogenates were analysed by western blot using lamin B1 and emerin antibodies and actin was visualised by ponceau red staining. A) Results expressed as the densitometric ratio of Lamin B1/Actin or Lamin B1/Emerin of *n* = 3 control and n = 3 DCM samples are shown. Each *n* represents a human myocardium tissue sample from a single donor. Bars represent the mean and standard deviation. Data was analysed by unpaired non-parametric *t-*test with Mann-Whitney corrections applied, *ns* non-significant. B) Lamin B1, emerin and actin western blot and ponceau red staining images used for the quantification in A. Control and donor IDs and diagnostic data are reported in Supplementary File 1. Human myocardium tissue homogenates were analysed by western blot using lamin B1 and emerin antibodies and actin was visualised by ponceau red staining. A) Results expressed as the densitometric ratio of Lamin B1/Actin or Lamin B1/Emerin of *n* = 3 control and n = 3 DCM samples are shown. Each *n* represents a human myocardium tissue sample from a single donor. Bars represent the mean and standard deviation. Data was analysed by unpaired non-parametric *t-*test with Mann-Whitney corrections applied, *ns* non-significant. B) Lamin B1, emerin and actin western blot and ponceau red staining images used for the quantification in A. Control and donor IDs and diagnostic data are reported in Supplementary File 1.

**Fig. 2:**
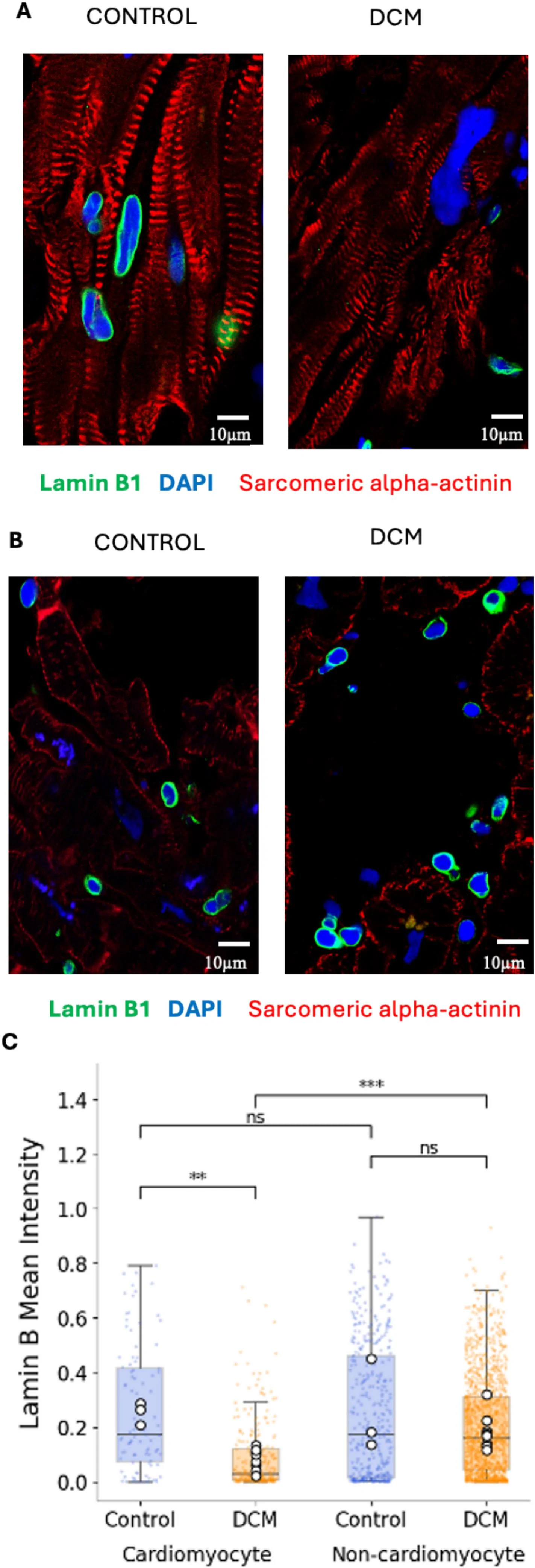
Lamin B1 quantification in human heart nuclei. Representative confocal images of immunofluorescence-stained sections of human myocardium: lamin B1 (green), DAPI (blue), and sarcomeric alpha-actinin (red). A) cardiomyocyte nuclei B) non-cardiomyocyte nuclei C) human heart nuclei lamin B1 quantification from immunofluorescence staining of myocardium sections from control non-failing (n = 3) and DCM (*n* = 8) donors. Small dots represent individual nuclei (cardiomyocyte: 95 control, 263 DCM; non-cardiomyocyte: 416 control, 1390 DCM; 2164 nuclei total from 79 fields of view). Large, white-outlined circles represent per-donor means. Boxes show the median and interquartile range. Significance was assessed by nested linear mixed-effects models fitted to nucleus-level data with donor and image (nested within donor) as random intercepts. *p*-Values were Holm–Bonferroni corrected across the four comparisons: cardiomyocyte control vs. DCM *p* = 0.0013; non-cardiomyocyte control vs. DCM *p* = 0.62; cardiomyocyte vs. non-cardiomyocyte in control *p* = 0.66; cardiomyocyte vs. non-cardiomyocyte in DCM *p* = 1.1 × 10^−4^. *p < 0.05, **p < 0.01, ***p < 0.001, ns = not significant. (For interpretation of the references to colour in this figure legend, the reader is referred to the web version of this article.)

We first used immunoblot analysis to quantify total lamin B1 expression in whole heart tissue homogenate in non-failing controls versus DCM samples. Lamin B1 intensity is normalised against actin from the Ponceau Red staining as whole protein content and emerin for nuclear lamina content. Although a small reduction of lamin B1 protein levels was observed in DCM samples compared to controls, this difference is not statistically significant (Fig. 1a-b). However, when immunofluorescence was followed by confocal microscopy of tissue samples, we observed a significant reduction of lamin B1 expression in DCM compared to non-failing cardiomyocyte nuclei. This reduction was localised to nuclei embedded within the cells expressing the sarcomeric *Z*-disc protein alpha-actinin and was not observed in other myocardial cells. This suggests that in DCM hearts, lamin B1 loss is specific to cardiomyocyte nuclei (Fig. 2a-c).

### 2.2 Morphological characterization of non-failing and DCM cardiomyocyte nuclei

**Fig. 3:**
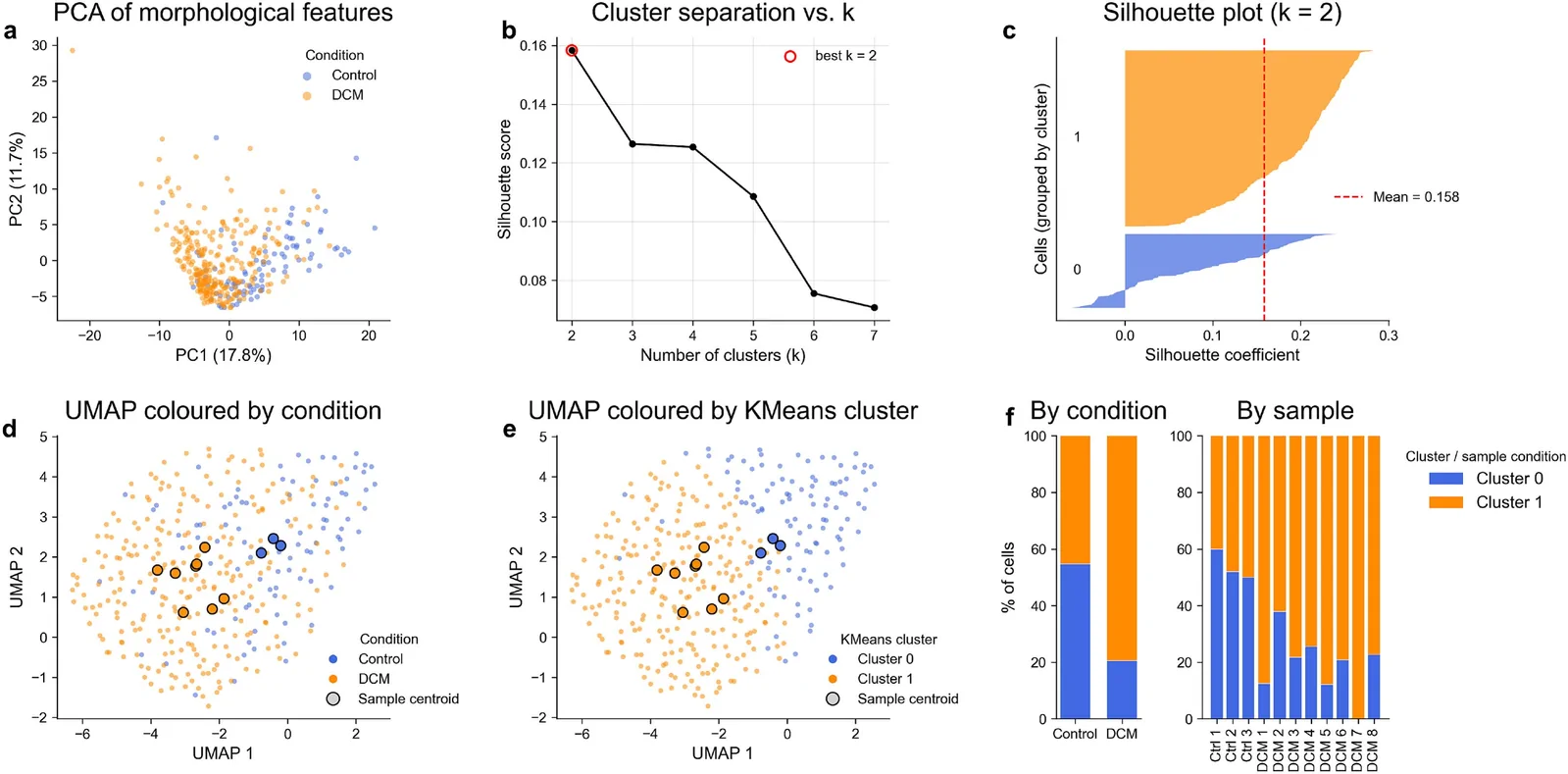
Dimensionality reduction and unsupervised clustering of cardiomyocytes features. A) Principal Component Analysis (PCA) of cardiomyocyte nuclei features. B) Silhouette score Cluster over number of clusters. C) Silhouette plot for 2 clusters. The dashed red line indicates the overall mean silhouette score of 0.157. D) UMAP projection coloured by condition. Small dots represent individual cells coloured by condition. Large dots indicate calculated sample centroids for individual replicates. E) UMAP projection coloured by cluster. Same UMAP embedding shown in (D) but coloured by cluster assignment. F) Cluster distribution by condition and biological sample. Stacked bar charts showing the percentage composition of cells assigned to Cluster 0 (blue) and Cluster 1 (orange). Left panel: proportional distribution aggregated by experimental condition (Control vs. DCM). Right panel: Proportional distribution by individual biological samples, with samples coloured by their respective cluster assignment. Control and donor IDs and diagnostic data are reported in Supplementary File 1. (For interpretation of the references to colour in this figure legend, the reader is referred to the web version of this article.) A) Principal Component Analysis (PCA) of cardiomyocyte nuclei features. B) Silhouette score Cluster over number of clusters. C) Silhouette plot for 2 clusters. The dashed red line indicates the overall mean silhouette score of 0.157. D) UMAP projection coloured by condition. Small dots represent individual cells coloured by condition. Large dots indicate calculated sample centroids for individual replicates. E) UMAP projection coloured by cluster. Same UMAP embedding shown in (D) but coloured by cluster assignment. F) Cluster distribution by condition and biological sample. Stacked bar charts showing the percentage composition of cells assigned to Cluster 0 (blue) and Cluster 1 (orange). Left panel: proportional distribution aggregated by experimental condition (Control vs. DCM). Right panel: Proportional distribution by individual biological samples, with samples coloured by their respective cluster assignment. Control and donor IDs and diagnostic data are reported in Supplementary File 1. (For interpretation of the references to colour in this figure legend, the reader is referred to the web version of this article.)

The immunofluorescence images suggest there is an increased prevalence of deformed nuclei with reduced lamin B1 expression in DCM-derived cardiomyocytes compared to donors (Fig. 2a). We therefore used unbiased features analysis of cardiomyocyte nuclei from donors to identify differences between non-failing and DCM donors. (Fig. 3a). We used CellProfiler to extract 192 morphological and intensity-based features from cardiomyocyte and non-cardiomyocyte nuclei. These features focus on nuclear size, shape and the intensity and distribution of lamin B1 [12] (Supplementary Table 1).

Principal Component Analysis (PCA) of cardiomyocyte nuclei features maps the cardiomyocytes into two partially overlapping regions based on whether they were from non-failing or DCM donors (Fig. 3a). This incomplete separation is explained by the observation that DCM hearts still contain a subpopulation of cardiomyocytes with non-deformed nuclei and normal lamin B1 levels. Similarly, in non-failing donor hearts, we can observe a few deformed nuclei with low lamin B1 content in cardiomyocytes.

**Fig. 4:**
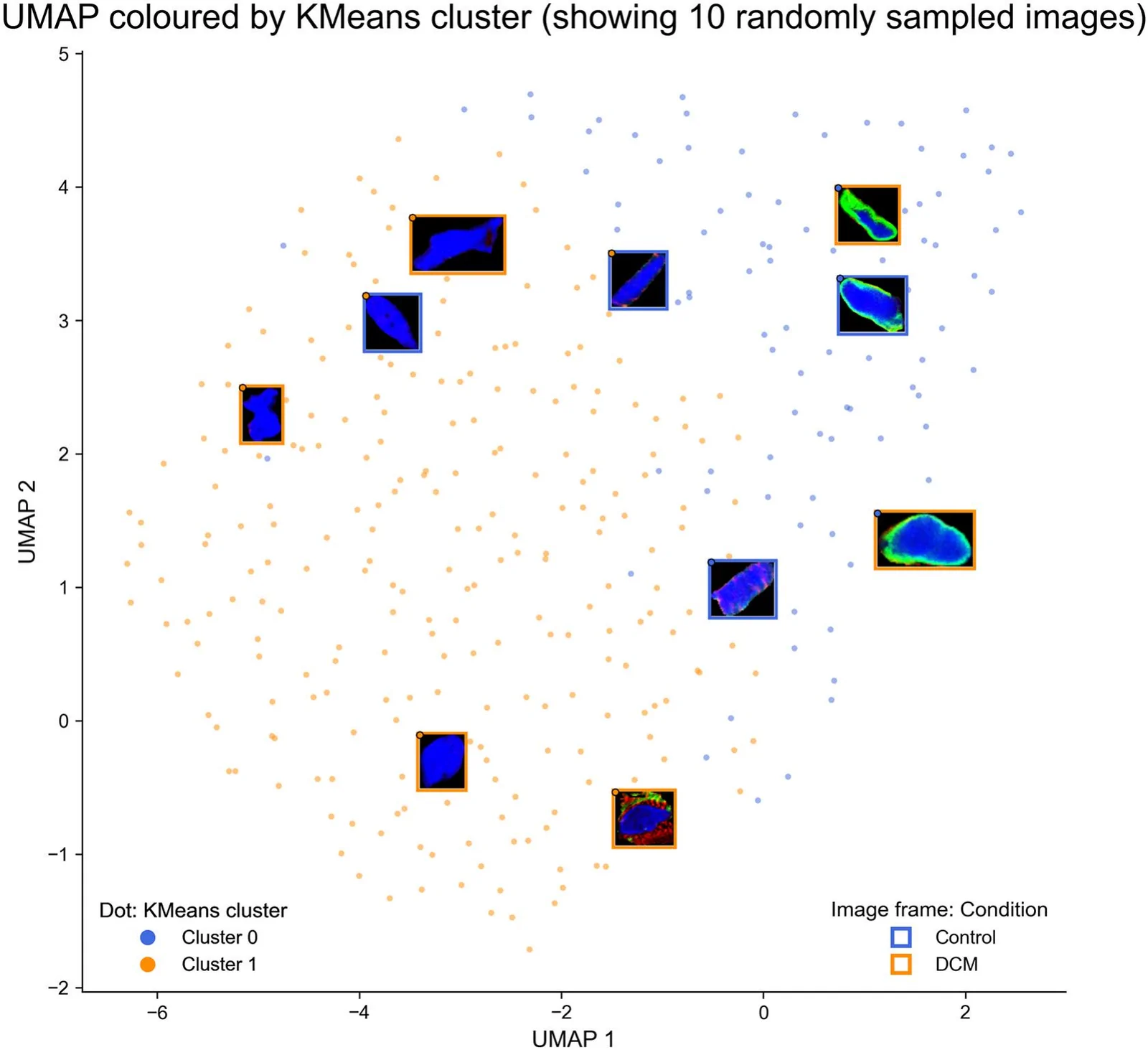
UMAP plot coloured by K-means cluster showing ten randomly samples images. UMAP projection coloured by cluster assignment (Cluster 0: blue Cluster 1: orange). Randomly selected images are shown at their corresponding location. The colour of images border indicates the sample origin (Blue: control and Orange: DCM). Displayed nuclei were randomly selected by setting np.random.default_rng(). (For interpretation of the references to colour in this figure legend, the reader is referred to the web version of this article.)

Consistently, K-means clustering identified two overlapping populations within the pooled dataset (Fig. 3b-c). When cross-referenced with the sample condition, nuclei from non-failing controls are predominantly enriched in cluster 1 whereas nuclei from DCM donors are enriched in cluster 0. Furthermore, plotting the calculated sample centroids on the Uniform Manifold Approximation and Projection (UMAP) projections (Fig. 3d-e) shows a clear spatial segregation between the non-failing and the DCM donors. Taken together, the data show a higher prevalence of cardiomyocytes with nuclear shape deformation and reduced levels of lamin B1 in DCM hearts. Specifically, this subpopulation (cluster 1: orange) constitutes 80.8% (±11.2%) of the cells in DCM donors, compared to 46% (±5.3%) in non-failing donors (Fig. 3f). Finally, when we display random selected images on the UMAP projection coloured by cluster assignment (Fig. 4), we observe that nuclear deformation and lamin B1 content correlate remarkably well with the position on the map and the non-failing-DCM classification. The more two images differ in lamin B1 content and nuclear shape, the further they are positioned on the UMAP projection.

## 3 Discussion

Nuclear lamin B1 integrity is crucial for the functional and structural health of cells. It provides structural integrity to the nucleus and regulates gene expression by restricting the accessibility to and transcription of lamin B1 tethered genes [13]. Disruption of lamin B1 architecture alters the expression profile of the affected cells in human brains and animal models of neurodegenerative diseases [14], [15], [16].

An increased prevalence of deformed nuclei and low lamin B1 levels is observed in cardiomyocytes from DCM hearts. We found a gradient between non-failing and diseased cardiomyocytes, where an accumulation of deformed nuclei with low lamin B1 levels is eventually associated with a DCM diagnosis*.* We also observed that the loss of lamin B1 is specific to cardiomyocytes and is not observed in other myocardial cells, such as the more numerous fibroblasts (Fig. 2a-c).

It is worth noting that our image analysis is unbiased. All cardiomyocyte nuclei images are pooled to feature extraction, and the non-failing/DCM classification is not used for the PCA and clustering analysis. It is therefore reassuring to observe that when plotting the calculated sample centroids on the UMAP projections there is a clear spatial segregation between the non-failing and DCM donors, showing that cardiomyocyte nuclei deformation and lamin B1 content correlate with the DCM diagnosis.

It is tempting to speculate, that karyoptosis, which was initially described in another postmitotic organ, the brain, might also be present in the heart. This is based on the observation that proteotoxic stress is one of the main drivers in both diseases, and that both neuron and cardiomyocyte nuclei display a loss of lamin B1 and nuclear deformation in the disease state. However, the data presented in this manuscript cannot answer whether karyoptosis occurs in the heart; further studies are needed to test if the signalling pathway leading to lamin B1 degradation (through MAPK p38 alpha activation) that was shown in the context of proteotoxic stress in neurons, is also present in cardiomyocytes.

This is the first study to describe an association between lamin B1 loss, nuclear deformation and a DCM diagnosis in human cardiomyocytes. The loss of lamin B1, given its role in chromatin architecture, is likely to modulate the transcriptional reprogramming observed in DCM cardiomyocytes [17], [18], [19]. However, our study only looks at a single timepoint (i.e. end stage of the disease at heart transplantation) and cannot confirm either a causal link between lamin B1 loss and nuclear shape deformation, or an effect on transcription. A further limitation of this study is the number of samples, we were limited in both the amount of sample received per donor (2 mm^3^) and the number of donors, this is therefore a pilot study and validation in larger number of samples will be needed in the future. Finally, it would be interesting to test if the nuclear features we observe in DCM are also present in other cardiomyopathies.

In conclusion, our study shows that lamin B1 loss in human cardiomyocytes is associated with nuclear shape deformation in DCM, however, no causal or mechanistic link can be inferred based on the data presented here.

## 4 Methods

### 4.1 Immunohistochemistry of human heart tissue

Human heart samples were procured and snap frozen within 15 min of harvest from donor hearts as previous described [20], [21], [22], [23], [24]. 10 μm sections were cut on a Leica CM1950 cryostat and retrieved onto Superfrost slides, allowed to air dry overnight and fixed in pre-chilled 100% acetone at −20 °C for 5 min, and then washed 2 × 5 min in phosphate-buffered saline (PBS). Sections were permeabilised in 0.2% Triton X-100 for 1 min at room temperature (RT) and washed 3 × 5 min in PBS. Sections were blocked in 5% preimmune goat serum (Sigma) in 1% bovine serum albumen (BSA)/Tris buffered Serum (TBS) for 1 h at RT. Primary antibody was diluted in 1% BSA/TBS applied to the sections and incubated in a humidified chamber overnight at 4 °C. Sections were then washed 3 × 5 min in PBS and the secondary antibody (conjugated to a fluorophore) was diluted in 1% BSA/TBS, applied to the sections and incubated in the dark in a humidified chamber for 1 h at RT. Slides were washed for 3 × 5 min and then embedded using Lisbeth's mounting medium. The primary antibody mixture was a combination of monoclonal rabbit anti-lamin B1 (abcam 229025) and was used together with the cell border markers beta-catenin and laminin (rabbit polyclonals; Sigma C2206; L9393) or vinculin (mouse monoclonal Sigma V9131) and monoclonal mouse anti-sarcomeric alpha-actinin (Sigma A7811) as published previously [23]. DAPI (Sigma) 0.1 μg/ml together with Cy3-conjugated goat anti-mouse immunoglobulin (multilabelling quality Jackson Immunochemicals 115-165-146) and Alexa488-conjugated goat anti-rabbit (multilabelling quality; Jackson Immunochemicals 111-545-144). Confocal microscopy was carried out using sequential scan mode on a Leica SP5 confocal, equipped with blue diode, argon- and helium‑neon lasers using a 63×/1.4NA oil-immersion lens. Images were exported as TIF files for downstream analysis.

### 4.2 Western blot analysis of cryopreserved heart tissue

Heart tissue slices, generated by a cryostat, were collected and lysed in buffer containing: 3.7 M urea, 134.6 mM Tris pH 6.8, 5.4% SDS, 2.3% NP-40, 4.45% β-mercaptoethanol, 4% glycerol, and 6 mg/100 ml bromophenol blue. Samples were heated to 100 °C for 2 min. Proteins were separated by SDS-PAGE on 4–20% precast gels (Generon) at 120 V. Transfer onto nitrocellulose membranes (G&E Healthcare) was carried out using wet blot technique at 65 mAmps overnight at 4 °C. Following visualisation of protein transfer using Ponceau Red (Sigma), membranes were blocked with 5% milk (Sainsbury's) in TBS-T for 1 h, followed by incubation with primary antibodies diluted in TBS-T for 1 h at RT. Primary antibodies as above, were used in addition to monoclonal mouse anti-emerin (abcam 54996). After 3 × 5 min washes in TBS-T, membranes were incubated with horse radish-conjugated secondary antibodies (Millipore 401315; DAKO P0260) for 1 h at room temperature. Signals were detected using Clarity (BioRad) and a BioRad Geldoc systems, followed by quantification using Fiji [25]. We have used the nuclear lamina protein emerin and the actin band from the Ponceau Red staining of the immunoblot, which includes all actin isoforms expressed, as our loading control for the quantifications of whole protein and nuclear lamina amount, respectively. Widely used loading controls such as GAPDH and beta-cytoplasmic actin are not useful for a metabolically active tissue like the heart, where the predominant actin isoform that is expressed is also alpha-cardiac actin.

### 4.3 Cardiomyocyte identification and classification analysis

#### 4.3.1 Single-cell segmentation and feature extraction

Individual cardiomyocyte nuclei were identified embedded in the striated pattern of the *Z*-disc protein sarcomeric alpha-actinin. For some images, vinculin or beta catenin labelling was also used to aid with the identification of the cardiomyocyte nuclei. Cardiomyocyte nuclei were manually segmented and a binary mask was created using Fiji [25] while non-cardyomyocyte nuclei were automatically segmented using CellProfiler (v4.2.8). The researcher manually segmenting the cardiomyocyte nuclei was blind to the donor classification of the samples. Single-nuclei features were extracted from the fluorescence images using CellProfiler. Each image was split into red, green and blue components (ColorToGray module). Cardiomyocyte binary masks were converted into labelled objects (ConvertImageToObjects), and all subsequent measurements were made on these single-cell objects using the green (lamin B1) channel as the intensity source. Non-cardiomyocyte objects were obtained by subtracting the cardiomyocyte masks to the whole image's objects. For each object, three measurement modules were applied to extract morphological and intensity-based features: MeasureObjectSizeShape, MeasureObjectIntensity and MeasureObjectIntensityDistribution. The description of the features used is reported in Supplementary Table 1. Single-cell crops were exported (SaveCroppedObjects) and the full per-object measurement table was written to disk (ExportToSpreadsheet). This per-cell feature table formed the input to the dimensionality reduction and clustering described below.

#### 4.3.2 Lamin B1 intensity analysis

Lamin B1 mean intensity per nucleus was quantified using CellProfiler as described above. Results were grouped by cell type (cardiomyocyte, non-cardiomyocyte) and condition (Control, DCM), giving four groups (*n* = 3 Control, *n* = 8 DCM donors, with 358 cardiomyocyte and 1806 non-cardiomyocyte nuclei; total = 2164). Normality was assessed per group by Shapiro-Wilk and D'Agostino K^2^ tests and variance homogeneity by Levene's test. All four groups departed from normality on raw and log₁₀-transformed values, and variances were unequal (Levene W = 62.7, *p* = 6.9 × 10^−39^).

As multiple nuclei were segmented per image and multiple images were acquired per donor, comparisons used nested linear mixed-effects models fitted to nucleus-level data with donor and image (nested within donor) as random intercepts (intensity ~ condition + (1 | donor) + (1 | image) within each cell type, and intensity ~ cell type + (1 | donor) + (1 | image) within each condition). Models were fitted by restricted maximum likelihood using the MixedLM class of statsmodels (statsmodels.formula.api.mixedlm), with donor supplied as the grouping factor, a donor random intercept specified via re_formula = “1”, and image entered as a variance component nested within donor via vc_formula. *p*-values were Holm–Bonferroni corrected across the four comparisons. Significance is indicated as *p < 0.05, **p < 0.01, ***p < 0.001, ns = not significant. Analyses were performed in Python 3.12 using pandas, NumPy, SciPy and statsmodels.

#### 4.3.3 Dimensionality reduction and clustering

Per-cell features were standardised and reduced by principal component analysis (PCA; scikit-learn). The first two principal components were used for visualisation, with the explained variance ratio reported for each. To assess the optimal number of clusters, k-means clustering was performed across a range of cluster numbers (k = 2–7), and clustering quality was evaluated using the mean silhouette coefficient computed on the PCA-transformed feature matrix. The configuration yielding the highest silhouette score was identified as optimal. Per-cluster silhouette coefficients were additionally examined for the k = 2 solution to assess within-cluster cohesion and separation.

#### 4.3.4 Visualisation by UMAP

Uniform Manifold Approximation and Projection (UMAP; umap-learn) was applied to the PCA-transformed features for two-dimensional embedding, using the parameter n_(neighbour = 30, min_dist = 0.5, a Euclidean distance metric), and a fixed random seed for reproducibility. Embeddings were coloured by experimental condition (Control, DCM) and by k-means cluster assignment. To summarise donor-level structure and account for inter-sample variability, per-sample centroids were computed as the mean UMAP coordinates of all cells from each donor and overlaid on the embeddings.

#### 4.3.5 Cluster composition analysis

The distribution of cells across clusters was quantified both by condition and by individual sample. For each grouping, cluster membership was expressed as the percentage of cells assigned to each cluster (normalised so that each condition or sample summed to 100%). Samples were ordered by condition to permit visual comparison of cluster composition between Control and DCM donors.

#### 4.3.6 Statistical software and figure generation

Statistical tests utilised are described in the figure legends and were carried out with GraphPad Prism software (v10.6.1) and Python using scikit-learn, umap-learn, pandas, NumPy, SciPy and statsmodels. Figures were generated with GraphPad Prism and Matplotlib and exported as png files.

## CRediT authorship contribution statement

**Leandro N. Ventimiglia:** Writing – review & editing, Writing – original draft, Methodology, Formal analysis, Data curation. **Aleksej Zelezniak:** Resources. **Cristobal G. dos Remedios:** Writing – review & editing. **Sean Lal:** Resources. **Elisabeth Ehler:** Writing – review & editing, Writing – original draft, Resources, Methodology. **Gian F. De Nicola:** Writing – review & editing, Writing – original draft, Supervision, Funding acquisition, Conceptualization.

## Ethical approval

The project has been approved by the Heart, Lung and Critical Care (HLCC) Access Committee and has therefore been included under the Ethical Approval given to the HLCC Biobank, NRES reference 24/SC/0342. Relevant human material and data was collected in accordance with the terms of ethical approval reference 24/SC/0342, and informed consent has been granted for the purposes of the project. Non-failing heart samples were obtained from the Sydney Heart Bank (Director C. dos Remedios, and now run by S. Lal) at the University of Sydney (Human Research Ethical Committee approval: 09-2009-12146).

## Declaration of competing interest

The authors declared no potential conflicts of interest with respect to the research, authorship, and publication of this article.

## Figures and Tables

**Supplementary Fig. 1:**
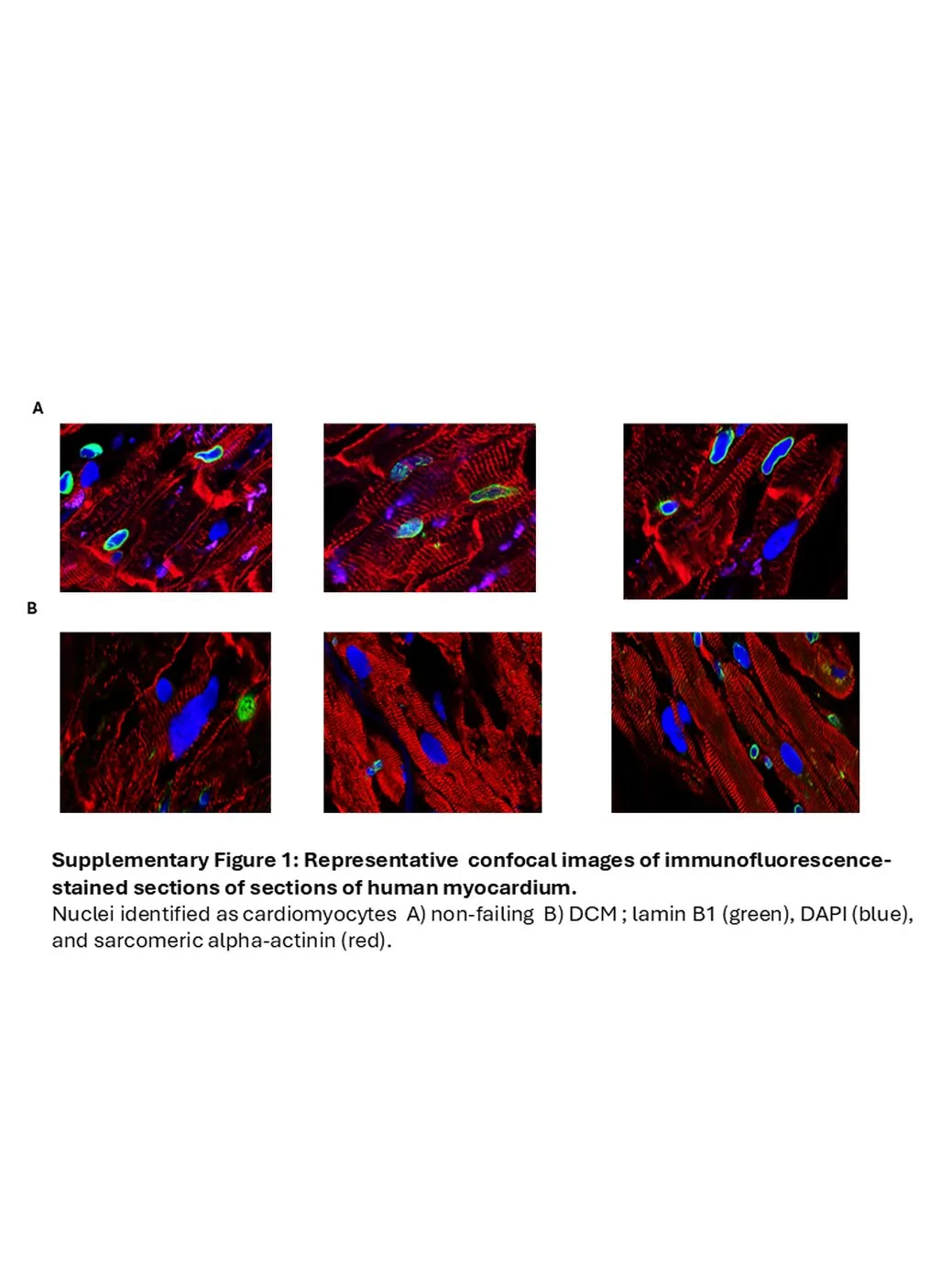
Representative confocal images of immunoflourescencestained sections of sections of human myocardium.

**Supplementary Fig. 2:**
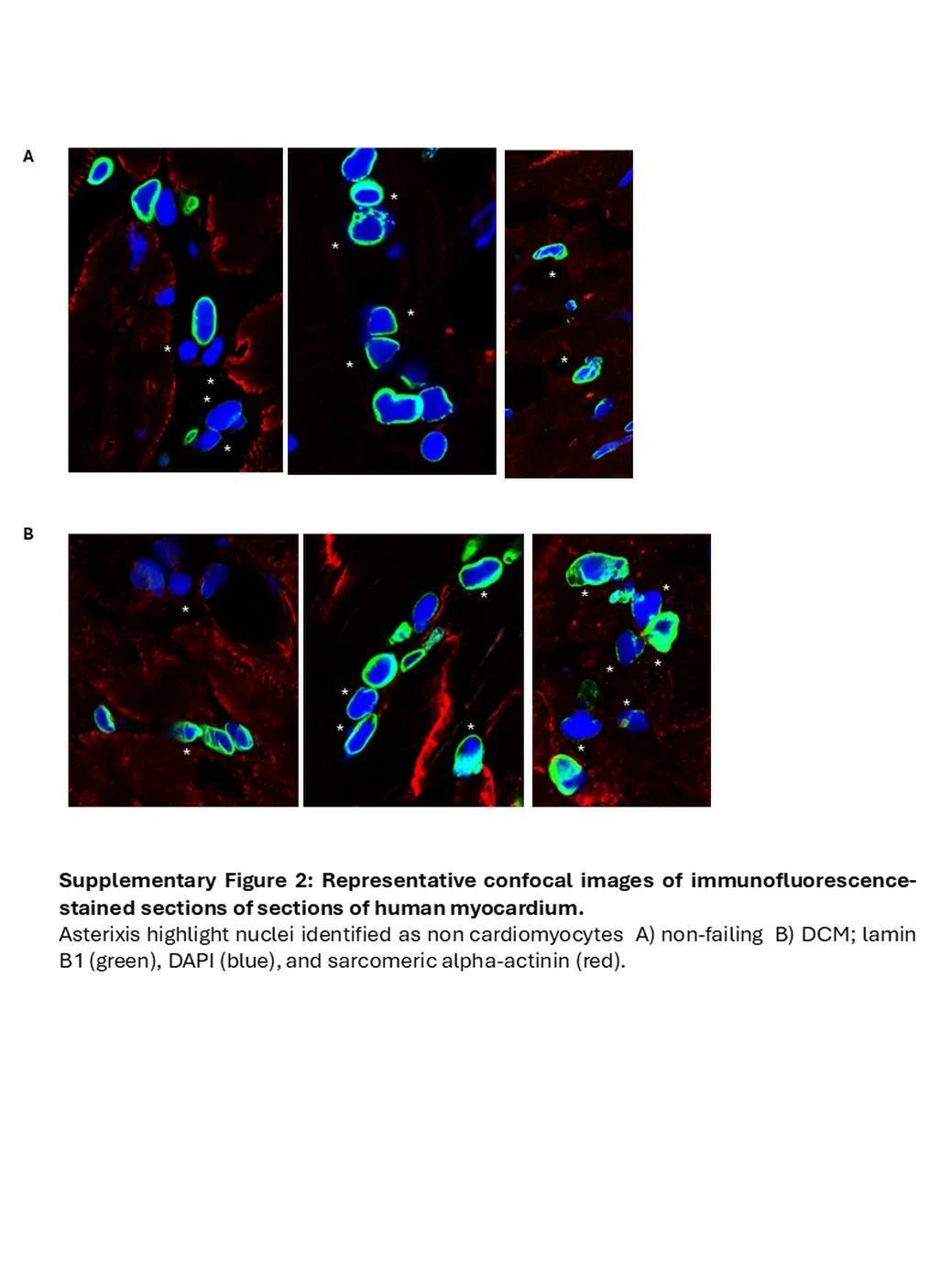
Representative confocal images of immunoflourescencestained sections of sections of human myocardium.

## Appendix A Supplementary data

Supplementary File 1 Excel file containing the available donor's data.

Supplementary material

Supplementary Table 1 CellProfiler feature definitions.
