# Supplementary material for: Lamin B1 loss in human cardiomyocytes is associated with nuclear shape deformation in dilated cardiomyopathy
Source: J Mol Cell Cardiol Plus. 2026 Aug 17;17:100865. doi: 10.1016/j.jmccpl.2026.100865 (PMC13524538; doi:10.1016/j.jmccpl.2026.100865)
Supplement: Supplementary file 1 [file mmc5.pdf]

Supplementary Table: CellProfiler feature definitions

Complete list of the 192 morphological and Lamin B1 intensity/distribution features extracted per nucleus using CellProfiler v4.2.8 (modules: MeasureObjectSizeShape, MeasureObjectIntensity, MeasureObjectIntensityDistribution). Definitions follow the official CellProfiler 4.2.8 module documentation.

Module	Feature	Definition
MeasureObjectSizeShape	**Area**	Number of pixels in the nucleus region.
MeasureObjectSizeShape	**Perimeter**	Total number of pixels around the boundary of the object.
MeasureObjectSizeShape	**ConvexArea**	Area of the convex hull (“rubber band” polygon) containing the object.
MeasureObjectSizeShape	**EquivalentDiameter**	Diameter of a circle with the same area as the object.
MeasureObjectSizeShape	**MajorAxisLength**	Length of the major axis of the ellipse with the same second moments as the object.
MeasureObjectSizeShape	**MinorAxisLength**	Length of the minor axis of the ellipse with the same second moments as the object.
MeasureObjectSizeShape	**MaxFeretDiameter**	Largest caliper diameter across all rotation angles.
MeasureObjectSizeShape	**MinFeretDiameter**	Smallest caliper diameter across all rotation angles.
MeasureObjectSizeShape	**MaximumRadius**	Maximum distance from any pixel inside the object to the nearest pixel outside it.
MeasureObjectSizeShape	**MeanRadius**	Mean distance from pixels inside the object to the nearest pixel outside it.
MeasureObjectSizeShape	**MedianRadius**	Median distance from pixels inside the object to the nearest pixel outside it.
MeasureObjectSizeShape	**BoundingBoxArea**	Area of the smallest axis-aligned box containing the object.
MeasureObjectSizeShape	**BoundingBoxMinimum_X**	Minimum x-coordinate of the object's bounding box.
MeasureObjectSizeShape	**BoundingBoxMinimum_Y**	Minimum y-coordinate of the object's bounding box.
MeasureObjectSizeShape	**BoundingBoxMaximum_X**	Maximum x-coordinate of the object's bounding box.
MeasureObjectSizeShape	**BoundingBoxMaximum_Y**	Maximum y-coordinate of the object's bounding box.
MeasureObjectSizeShape	**Center_X**	X-coordinate of the object centroid (point farthest from any edge).
MeasureObjectSizeShape	**Center_Y**	Y-coordinate of the object centroid (point farthest from any edge).
MeasureObjectSizeShape	**Solidity**	Proportion of convex-hull pixels also inside the object (Area / ConvexArea).
MeasureObjectSizeShape	**Extent**	Proportion of the bounding box occupied by the object.
MeasureObjectSizeShape	**FormFactor**	4 x pi x Area / Perimeter^2; equals 1 for a perfectly circular object.
MeasureObjectSizeShape	**Compactness**	Perimeter^2 / (4 x pi x Area); a filled circle has a value of 1, irregular objects score higher.
MeasureObjectSizeShape	**Eccentricity**	Eccentricity of the ellipse with the same second moments as the object (0 = circle, 1 = line segment).
MeasureObjectSizeShape	**Orientation**	Angle (–90° to 90°) between the x-axis and the major axis of the equivalent ellipse.
MeasureObjectSizeShape	**EulerNumber**	Number of objects minus number of holes in the region, assuming 8-connectivity.
MeasureObjectSizeShape	**Zernike_0_0**	Zernike shape moment of order 0, degree 0 — coefficient describing the object's outline shape in a basis of Zernike polynomials (orders 0–9 calculated).
MeasureObjectSizeShape	**Zernike_1_1**	Zernike shape moment of order 1, degree 1 — coefficient describing the object's outline shape in a basis of Zernike polynomials (orders 0–9 calculated).
MeasureObjectSizeShape	**Zernike_2_0**	Zernike shape moment of order 2, degree 0 — coefficient describing the object's outline shape in a basis of Zernike polynomials (orders 0–9 calculated).
MeasureObjectSizeShape	**Zernike_2_2**	Zernike shape moment of order 2, degree 2 — coefficient describing the object's outline shape in a basis of Zernike polynomials (orders 0–9 calculated).
MeasureObjectSizeShape	**Zernike_3_1**	Zernike shape moment of order 3, degree 1 — coefficient describing the object's outline shape in a basis of Zernike polynomials (orders 0–9 calculated).

Module	Feature	Definition
MeasureObjectSizeShape	**Zernike_3_3**	Zernike shape moment of order 3, degree 3 — coefficient describing the object's outline shape in a basis of Zernike polynomials (orders 0–9 calculated).
MeasureObjectSizeShape	**Zernike_4_0**	Zernike shape moment of order 4, degree 0 — coefficient describing the object's outline shape in a basis of Zernike polynomials (orders 0–9 calculated).
MeasureObjectSizeShape	**Zernike_4_2**	Zernike shape moment of order 4, degree 2 — coefficient describing the object's outline shape in a basis of Zernike polynomials (orders 0–9 calculated).
MeasureObjectSizeShape	**Zernike_4_4**	Zernike shape moment of order 4, degree 4 — coefficient describing the object's outline shape in a basis of Zernike polynomials (orders 0–9 calculated).
MeasureObjectSizeShape	**Zernike_5_1**	Zernike shape moment of order 5, degree 1 — coefficient describing the object's outline shape in a basis of Zernike polynomials (orders 0–9 calculated).
MeasureObjectSizeShape	**Zernike_5_3**	Zernike shape moment of order 5, degree 3 — coefficient describing the object's outline shape in a basis of Zernike polynomials (orders 0–9 calculated).
MeasureObjectSizeShape	**Zernike_5_5**	Zernike shape moment of order 5, degree 5 — coefficient describing the object's outline shape in a basis of Zernike polynomials (orders 0–9 calculated).
MeasureObjectSizeShape	**Zernike_6_0**	Zernike shape moment of order 6, degree 0 — coefficient describing the object's outline shape in a basis of Zernike polynomials (orders 0–9 calculated).
MeasureObjectSizeShape	**Zernike_6_2**	Zernike shape moment of order 6, degree 2 — coefficient describing the object's outline shape in a basis of Zernike polynomials (orders 0–9 calculated).
MeasureObjectSizeShape	**Zernike_6_4**	Zernike shape moment of order 6, degree 4 — coefficient describing the object's outline shape in a basis of Zernike polynomials (orders 0–9 calculated).
MeasureObjectSizeShape	**Zernike_6_6**	Zernike shape moment of order 6, degree 6 — coefficient describing the object's outline shape in a basis of Zernike polynomials (orders 0–9 calculated).
MeasureObjectSizeShape	**Zernike_7_1**	Zernike shape moment of order 7, degree 1 — coefficient describing the object's outline shape in a basis of Zernike polynomials (orders 0–9 calculated).
MeasureObjectSizeShape	**Zernike_7_3**	Zernike shape moment of order 7, degree 3 — coefficient describing the object's outline shape in a basis of Zernike polynomials (orders 0–9 calculated).
MeasureObjectSizeShape	**Zernike_7_5**	Zernike shape moment of order 7, degree 5 — coefficient describing the object's outline shape in a basis of Zernike polynomials (orders 0–9 calculated).
MeasureObjectSizeShape	**Zernike_7_7**	Zernike shape moment of order 7, degree 7 — coefficient describing the object's outline shape in a basis of Zernike polynomials (orders 0–9 calculated).
MeasureObjectSizeShape	**Zernike_8_0**	Zernike shape moment of order 8, degree 0 — coefficient describing the object's outline shape in a basis of Zernike polynomials (orders 0–9 calculated).
MeasureObjectSizeShape	**Zernike_8_2**	Zernike shape moment of order 8, degree 2 — coefficient describing the object's outline shape in a basis of Zernike polynomials (orders 0–9 calculated).
MeasureObjectSizeShape	**Zernike_8_4**	Zernike shape moment of order 8, degree 4 — coefficient describing the object's outline shape in a basis of Zernike polynomials (orders 0–9 calculated).
MeasureObjectSizeShape	**Zernike_8_6**	Zernike shape moment of order 8, degree 6 — coefficient describing the object's outline shape in a basis of Zernike polynomials (orders 0–9 calculated).
MeasureObjectSizeShape	**Zernike_8_8**	Zernike shape moment of order 8, degree 8 — coefficient describing the object's outline shape in a basis of Zernike polynomials (orders 0–9 calculated).
MeasureObjectSizeShape	**Zernike_9_1**	Zernike shape moment of order 9, degree 1 — coefficient describing the object's outline shape in a basis of Zernike polynomials (orders 0–9 calculated).

Module	Feature	Definition
MeasureObjectSizeShape	**Zernike_9_3**	Zernike shape moment of order 9, degree 3 — coefficient describing the object's outline shape in a basis of Zernike polynomials (orders 0–9 calculated).
MeasureObjectSizeShape	**Zernike_9_5**	Zernike shape moment of order 9, degree 5 — coefficient describing the object's outline shape in a basis of Zernike polynomials (orders 0–9 calculated).
MeasureObjectSizeShape	**Zernike_9_7**	Zernike shape moment of order 9, degree 7 — coefficient describing the object's outline shape in a basis of Zernike polynomials (orders 0–9 calculated).
MeasureObjectSizeShape	**Zernike_9_9**	Zernike shape moment of order 9, degree 9 — coefficient describing the object's outline shape in a basis of Zernike polynomials (orders 0–9 calculated).
MeasureObjectSizeShape	**HuMoment_0**	Hu invariant image moment #0 — shape descriptor invariant to translation, scale, and rotation.
MeasureObjectSizeShape	**HuMoment_1**	Hu invariant image moment #1 — shape descriptor invariant to translation, scale, and rotation.
MeasureObjectSizeShape	**HuMoment_2**	Hu invariant image moment #2 — shape descriptor invariant to translation, scale, and rotation.
MeasureObjectSizeShape	**HuMoment_3**	Hu invariant image moment #3 — shape descriptor invariant to translation, scale, and rotation.
MeasureObjectSizeShape	**HuMoment_4**	Hu invariant image moment #4 — shape descriptor invariant to translation, scale, and rotation.
MeasureObjectSizeShape	**HuMoment_5**	Hu invariant image moment #5 — shape descriptor invariant to translation, scale, and rotation.
MeasureObjectSizeShape	**HuMoment_6**	Hu invariant image moment #6 — shape descriptor invariant to translation, scale, and rotation.
MeasureObjectSizeShape	**SpatialMoment_0_0**	Weighted-average (raw) image moment describing the object's shape, size, rotation, and location.
MeasureObjectSizeShape	**SpatialMoment_0_1**	Weighted-average (raw) image moment describing the object's shape, size, rotation, and location.
MeasureObjectSizeShape	**SpatialMoment_0_2**	Weighted-average (raw) image moment describing the object's shape, size, rotation, and location.
MeasureObjectSizeShape	**SpatialMoment_0_3**	Weighted-average (raw) image moment describing the object's shape, size, rotation, and location.
MeasureObjectSizeShape	**SpatialMoment_1_0**	Weighted-average (raw) image moment describing the object's shape, size, rotation, and location.
MeasureObjectSizeShape	**SpatialMoment_1_1**	Weighted-average (raw) image moment describing the object's shape, size, rotation, and location.
MeasureObjectSizeShape	**SpatialMoment_1_2**	Weighted-average (raw) image moment describing the object's shape, size, rotation, and location.
MeasureObjectSizeShape	**SpatialMoment_1_3**	Weighted-average (raw) image moment describing the object's shape, size, rotation, and location.
MeasureObjectSizeShape	**SpatialMoment_2_0**	Weighted-average (raw) image moment describing the object's shape, size, rotation, and location.
MeasureObjectSizeShape	**SpatialMoment_2_1**	Weighted-average (raw) image moment describing the object's shape, size, rotation, and location.
MeasureObjectSizeShape	**SpatialMoment_2_2**	Weighted-average (raw) image moment describing the object's shape, size, rotation, and location.
MeasureObjectSizeShape	**SpatialMoment_2_3**	Weighted-average (raw) image moment describing the object's shape, size, rotation, and location.
MeasureObjectSizeShape	**CentralMoment_0_0**	Spatial moment normalized to the object's centroid, making it invariant to the object's location in the image.
MeasureObjectSizeShape	**CentralMoment_0_1**	Spatial moment normalized to the object's centroid, making it invariant to the object's location in the image.
MeasureObjectSizeShape	**CentralMoment_0_2**	Spatial moment normalized to the object's centroid, making it invariant to the object's location in the image.
MeasureObjectSizeShape	**CentralMoment_0_3**	Spatial moment normalized to the object's centroid, making it invariant to the object's location in the image.

Module	Feature	Definition
MeasureObjectSizeShape	**CentralMoment_1_0**	Spatial moment normalized to the object's centroid, making it invariant to the object's location in the image.
MeasureObjectSizeShape	**CentralMoment_1_1**	Spatial moment normalized to the object's centroid, making it invariant to the object's location in the image.
MeasureObjectSizeShape	**CentralMoment_1_2**	Spatial moment normalized to the object's centroid, making it invariant to the object's location in the image.
MeasureObjectSizeShape	**CentralMoment_1_3**	Spatial moment normalized to the object's centroid, making it invariant to the object's location in the image.
MeasureObjectSizeShape	**CentralMoment_2_0**	Spatial moment normalized to the object's centroid, making it invariant to the object's location in the image.
MeasureObjectSizeShape	**CentralMoment_2_1**	Spatial moment normalized to the object's centroid, making it invariant to the object's location in the image.
MeasureObjectSizeShape	**CentralMoment_2_2**	Spatial moment normalized to the object's centroid, making it invariant to the object's location in the image.
MeasureObjectSizeShape	**CentralMoment_2_3**	Spatial moment normalized to the object's centroid, making it invariant to the object's location in the image.
MeasureObjectSizeShape	**NormalizedMoment_0_2**	Central moment further normalized to be scale-invariant, in addition to being location-invariant.
MeasureObjectSizeShape	**NormalizedMoment_0_3**	Central moment further normalized to be scale-invariant, in addition to being location-invariant.
MeasureObjectSizeShape	**NormalizedMoment_1_1**	Central moment further normalized to be scale-invariant, in addition to being location-invariant.
MeasureObjectSizeShape	**NormalizedMoment_1_2**	Central moment further normalized to be scale-invariant, in addition to being location-invariant.
MeasureObjectSizeShape	**NormalizedMoment_1_3**	Central moment further normalized to be scale-invariant, in addition to being location-invariant.
MeasureObjectSizeShape	**NormalizedMoment_2_0**	Central moment further normalized to be scale-invariant, in addition to being location-invariant.
MeasureObjectSizeShape	**NormalizedMoment_2_1**	Central moment further normalized to be scale-invariant, in addition to being location-invariant.
MeasureObjectSizeShape	**NormalizedMoment_2_2**	Central moment further normalized to be scale-invariant, in addition to being location-invariant.
MeasureObjectSizeShape	**NormalizedMoment_2_3**	Central moment further normalized to be scale-invariant, in addition to being location-invariant.
MeasureObjectSizeShape	**NormalizedMoment_3_0**	Central moment further normalized to be scale-invariant, in addition to being location-invariant.
MeasureObjectSizeShape	**NormalizedMoment_3_1**	Central moment further normalized to be scale-invariant, in addition to being location-invariant.
MeasureObjectSizeShape	**NormalizedMoment_3_2**	Central moment further normalized to be scale-invariant, in addition to being location-invariant.
MeasureObjectSizeShape	**NormalizedMoment_3_3**	Central moment further normalized to be scale-invariant, in addition to being location-invariant.
MeasureObjectSizeShape	**InertiaTensor_0_0**	Component of the rotational inertia tensor of the object, computed relative to its center.
MeasureObjectSizeShape	**InertiaTensor_0_1**	Component of the rotational inertia tensor of the object, computed relative to its center.
MeasureObjectSizeShape	**InertiaTensor_1_0**	Component of the rotational inertia tensor of the object, computed relative to its center.
MeasureObjectSizeShape	**InertiaTensor_1_1**	Component of the rotational inertia tensor of the object, computed relative to its center.
MeasureObjectSizeShape	**InertiaTensorEigenvalues_0**	Eigenvalue #0 of the object's inertia tensor, describing the distribution of mass around the principal axes.
MeasureObjectSizeShape	**InertiaTensorEigenvalues_1**	Eigenvalue #1 of the object's inertia tensor, describing the distribution of mass around the principal axes.
MeasureObjectIntensity	**IntegratedIntensity_OrigGreen**	Sum of Lamin B1 pixel intensities within the nucleus.
MeasureObjectIntensity	**MeanIntensity_OrigGreen**	Average Lamin B1 pixel intensity within the nucleus.
MeasureObjectIntensity	**MedianIntensity_OrigGreen**	Median Lamin B1 pixel intensity within the nucleus.

Module	Feature	Definition
MeasureObjectIntensity	**StdIntensity_OrigGreen**	Standard deviation of Lamin B1 pixel intensities within the nucleus.
MeasureObjectIntensity	**MADIntensity_OrigGreen**	Median absolute deviation of Lamin B1 intensities within the nucleus; MAD = median(|xi - median(x)|).
MeasureObjectIntensity	**MinIntensity_OrigGreen**	Minimum Lamin B1 pixel intensity within the nucleus.
MeasureObjectIntensity	**MaxIntensity_OrigGreen**	Maximum Lamin B1 pixel intensity within the nucleus.
MeasureObjectIntensity	**LowerQuartileIntensity_OrigGreen**	Lamin B1 intensity value below which 25% of the nucleus's pixels fall.
MeasureObjectIntensity	**UpperQuartileIntensity_OrigGreen**	Lamin B1 intensity value below which 75% of the nucleus's pixels fall.
MeasureObjectIntensity	**IntegratedIntensityEdge_OrigGreen**	Sum of Lamin B1 pixel intensities along the nucleus boundary only.
MeasureObjectIntensity	**MeanIntensityEdge_OrigGreen**	Average Lamin B1 pixel intensity along the nucleus boundary only.
MeasureObjectIntensity	**StdIntensityEdge_OrigGreen**	Standard deviation of Lamin B1 intensity along the nucleus boundary only.
MeasureObjectIntensity	**MinIntensityEdge_OrigGreen**	Minimum Lamin B1 pixel intensity along the nucleus boundary only.
MeasureObjectIntensity	**MaxIntensityEdge_OrigGreen**	Maximum Lamin B1 pixel intensity along the nucleus boundary only.
MeasureObjectIntensity	**MassDisplacement_OrigGreen**	Distance between the intensity-weighted centroid and the binary-mask centroid of the nucleus.
MeasureObjectIntensityDistribution	**FracAtD_OrigGreen_1of4**	Fraction of total Lamin B1 signal located within radial bin 1 of 4 (center to edge).
MeasureObjectIntensityDistribution	**FracAtD_OrigGreen_2of4**	Fraction of total Lamin B1 signal located within radial bin 2 of 4 (center to edge).
MeasureObjectIntensityDistribution	**FracAtD_OrigGreen_3of4**	Fraction of total Lamin B1 signal located within radial bin 3 of 4 (center to edge).
MeasureObjectIntensityDistribution	**FracAtD_OrigGreen_4of4**	Fraction of total Lamin B1 signal located within radial bin 4 of 4 (center to edge).
MeasureObjectIntensityDistribution	**MeanFrac_OrigGreen_1of4**	Fractional Lamin B1 intensity in radial bin 1 of 4, normalized by the fraction of nuclear pixels in that bin.
MeasureObjectIntensityDistribution	**MeanFrac_OrigGreen_2of4**	Fractional Lamin B1 intensity in radial bin 2 of 4, normalized by the fraction of nuclear pixels in that bin.
MeasureObjectIntensityDistribution	**MeanFrac_OrigGreen_3of4**	Fractional Lamin B1 intensity in radial bin 3 of 4, normalized by the fraction of nuclear pixels in that bin.
MeasureObjectIntensityDistribution	**MeanFrac_OrigGreen_4of4**	Fractional Lamin B1 intensity in radial bin 4 of 4, normalized by the fraction of nuclear pixels in that bin.
MeasureObjectIntensityDistribution	**RadialCV_OrigGreen_1of4**	Coefficient of variation of Lamin B1 intensity within radial bin 1 of 4, computed across 8 angular slices (captures angular asymmetry).
MeasureObjectIntensityDistribution	**RadialCV_OrigGreen_2of4**	Coefficient of variation of Lamin B1 intensity within radial bin 2 of 4, computed across 8 angular slices (captures angular asymmetry).
MeasureObjectIntensityDistribution	**RadialCV_OrigGreen_3of4**	Coefficient of variation of Lamin B1 intensity within radial bin 3 of 4, computed across 8 angular slices (captures angular asymmetry).
MeasureObjectIntensityDistribution	**RadialCV_OrigGreen_4of4**	Coefficient of variation of Lamin B1 intensity within radial bin 4 of 4, computed across 8 angular slices (captures angular asymmetry).
MeasureObjectIntensityDistribution	**ZernikeMagnitude_OrigGreen_0_0**	Rotation-invariant magnitude of the order-0, degree-0 Zernike moment of the Lamin B1 intensity distribution within the nucleus.
MeasureObjectIntensityDistribution	**ZernikeMagnitude_OrigGreen_1_1**	Rotation-invariant magnitude of the order-1, degree-1 Zernike moment of the Lamin B1 intensity distribution within the nucleus.
MeasureObjectIntensityDistribution	**ZernikeMagnitude_OrigGreen_2_0**	Rotation-invariant magnitude of the order-2, degree-0 Zernike moment of the Lamin B1 intensity distribution within the nucleus.
MeasureObjectIntensityDistribution	**ZernikeMagnitude_OrigGreen_2_2**	Rotation-invariant magnitude of the order-2, degree-2 Zernike moment of the Lamin B1 intensity distribution within the nucleus.
MeasureObjectIntensityDistribution	**ZernikeMagnitude_OrigGreen_3_1**	Rotation-invariant magnitude of the order-3, degree-1 Zernike moment of the Lamin B1 intensity distribution within the nucleus.
MeasureObjectIntensityDistribution	**ZernikeMagnitude_OrigGreen_3_3**	Rotation-invariant magnitude of the order-3, degree-3 Zernike moment of the Lamin B1 intensity distribution within the nucleus.
MeasureObjectIntensityDistribution	**ZernikeMagnitude_OrigGreen_4_0**	Rotation-invariant magnitude of the order-4, degree-0 Zernike moment of the Lamin B1 intensity distribution within the nucleus.
MeasureObjectIntensityDistribution	**ZernikeMagnitude_OrigGreen_4_2**	Rotation-invariant magnitude of the order-4, degree-2 Zernike moment of the Lamin B1 intensity distribution within the nucleus.

Module	Feature	Definition
MeasureObjectIntensityDistribution	**ZernikeMagnitude_OrigGreen_4_4**	Rotation-invariant magnitude of the order-4, degree-4 Zernike moment of the Lamin B1 intensity distribution within the nucleus.
MeasureObjectIntensityDistribution	**ZernikeMagnitude_OrigGreen_5_1**	Rotation-invariant magnitude of the order-5, degree-1 Zernike moment of the Lamin B1 intensity distribution within the nucleus.
MeasureObjectIntensityDistribution	**ZernikeMagnitude_OrigGreen_5_3**	Rotation-invariant magnitude of the order-5, degree-3 Zernike moment of the Lamin B1 intensity distribution within the nucleus.
MeasureObjectIntensityDistribution	**ZernikeMagnitude_OrigGreen_5_5**	Rotation-invariant magnitude of the order-5, degree-5 Zernike moment of the Lamin B1 intensity distribution within the nucleus.
MeasureObjectIntensityDistribution	**ZernikeMagnitude_OrigGreen_6_0**	Rotation-invariant magnitude of the order-6, degree-0 Zernike moment of the Lamin B1 intensity distribution within the nucleus.
MeasureObjectIntensityDistribution	**ZernikeMagnitude_OrigGreen_6_2**	Rotation-invariant magnitude of the order-6, degree-2 Zernike moment of the Lamin B1 intensity distribution within the nucleus.
MeasureObjectIntensityDistribution	**ZernikeMagnitude_OrigGreen_6_4**	Rotation-invariant magnitude of the order-6, degree-4 Zernike moment of the Lamin B1 intensity distribution within the nucleus.
MeasureObjectIntensityDistribution	**ZernikeMagnitude_OrigGreen_6_6**	Rotation-invariant magnitude of the order-6, degree-6 Zernike moment of the Lamin B1 intensity distribution within the nucleus.
MeasureObjectIntensityDistribution	**ZernikeMagnitude_OrigGreen_7_1**	Rotation-invariant magnitude of the order-7, degree-1 Zernike moment of the Lamin B1 intensity distribution within the nucleus.
MeasureObjectIntensityDistribution	**ZernikeMagnitude_OrigGreen_7_3**	Rotation-invariant magnitude of the order-7, degree-3 Zernike moment of the Lamin B1 intensity distribution within the nucleus.
MeasureObjectIntensityDistribution	**ZernikeMagnitude_OrigGreen_7_5**	Rotation-invariant magnitude of the order-7, degree-5 Zernike moment of the Lamin B1 intensity distribution within the nucleus.
MeasureObjectIntensityDistribution	**ZernikeMagnitude_OrigGreen_7_7**	Rotation-invariant magnitude of the order-7, degree-7 Zernike moment of the Lamin B1 intensity distribution within the nucleus.
MeasureObjectIntensityDistribution	**ZernikeMagnitude_OrigGreen_8_0**	Rotation-invariant magnitude of the order-8, degree-0 Zernike moment of the Lamin B1 intensity distribution within the nucleus.
MeasureObjectIntensityDistribution	**ZernikeMagnitude_OrigGreen_8_2**	Rotation-invariant magnitude of the order-8, degree-2 Zernike moment of the Lamin B1 intensity distribution within the nucleus.
MeasureObjectIntensityDistribution	**ZernikeMagnitude_OrigGreen_8_4**	Rotation-invariant magnitude of the order-8, degree-4 Zernike moment of the Lamin B1 intensity distribution within the nucleus.
MeasureObjectIntensityDistribution	**ZernikeMagnitude_OrigGreen_8_6**	Rotation-invariant magnitude of the order-8, degree-6 Zernike moment of the Lamin B1 intensity distribution within the nucleus.
MeasureObjectIntensityDistribution	**ZernikeMagnitude_OrigGreen_8_8**	Rotation-invariant magnitude of the order-8, degree-8 Zernike moment of the Lamin B1 intensity distribution within the nucleus.
MeasureObjectIntensityDistribution	**ZernikeMagnitude_OrigGreen_9_1**	Rotation-invariant magnitude of the order-9, degree-1 Zernike moment of the Lamin B1 intensity distribution within the nucleus.
MeasureObjectIntensityDistribution	**ZernikeMagnitude_OrigGreen_9_3**	Rotation-invariant magnitude of the order-9, degree-3 Zernike moment of the Lamin B1 intensity distribution within the nucleus.
MeasureObjectIntensityDistribution	**ZernikeMagnitude_OrigGreen_9_5**	Rotation-invariant magnitude of the order-9, degree-5 Zernike moment of the Lamin B1 intensity distribution within the nucleus.
MeasureObjectIntensityDistribution	**ZernikeMagnitude_OrigGreen_9_7**	Rotation-invariant magnitude of the order-9, degree-7 Zernike moment of the Lamin B1 intensity distribution within the nucleus.
MeasureObjectIntensityDistribution	**ZernikeMagnitude_OrigGreen_9_9**	Rotation-invariant magnitude of the order-9, degree-9 Zernike moment of the Lamin B1 intensity distribution within the nucleus.
MeasureObjectIntensityDistribution	**ZernikePhase_OrigGreen_0_0**	Orientation (phase) of the order-0, degree-0 Zernike moment of the Lamin B1 intensity distribution within the nucleus.
MeasureObjectIntensityDistribution	**ZernikePhase_OrigGreen_1_1**	Orientation (phase) of the order-1, degree-1 Zernike moment of the Lamin B1 intensity distribution within the nucleus.
MeasureObjectIntensityDistribution	**ZernikePhase_OrigGreen_2_0**	Orientation (phase) of the order-2, degree-0 Zernike moment of the Lamin B1 intensity distribution within the nucleus.
MeasureObjectIntensityDistribution	**ZernikePhase_OrigGreen_2_2**	Orientation (phase) of the order-2, degree-2 Zernike moment of the Lamin B1 intensity distribution within the nucleus.
MeasureObjectIntensityDistribution	**ZernikePhase_OrigGreen_3_1**	Orientation (phase) of the order-3, degree-1 Zernike moment of the Lamin B1 intensity distribution within the nucleus.
MeasureObjectIntensityDistribution	**ZernikePhase_OrigGreen_3_3**	Orientation (phase) of the order-3, degree-3 Zernike moment of the Lamin B1 intensity distribution within the nucleus.
MeasureObjectIntensityDistribution	**ZernikePhase_OrigGreen_4_0**	Orientation (phase) of the order-4, degree-0 Zernike moment of the Lamin B1 intensity distribution within the nucleus.

Module	Feature	Definition
MeasureObjectIntensityDistribution	**ZernikePhase_OrigGreen_4_2**	Orientation (phase) of the order-4, degree-2 Zernike moment of the Lamin B1 intensity distribution within the nucleus.
MeasureObjectIntensityDistribution	**ZernikePhase_OrigGreen_4_4**	Orientation (phase) of the order-4, degree-4 Zernike moment of the Lamin B1 intensity distribution within the nucleus.
MeasureObjectIntensityDistribution	**ZernikePhase_OrigGreen_5_1**	Orientation (phase) of the order-5, degree-1 Zernike moment of the Lamin B1 intensity distribution within the nucleus.
MeasureObjectIntensityDistribution	**ZernikePhase_OrigGreen_5_3**	Orientation (phase) of the order-5, degree-3 Zernike moment of the Lamin B1 intensity distribution within the nucleus.
MeasureObjectIntensityDistribution	**ZernikePhase_OrigGreen_5_5**	Orientation (phase) of the order-5, degree-5 Zernike moment of the Lamin B1 intensity distribution within the nucleus.
MeasureObjectIntensityDistribution	**ZernikePhase_OrigGreen_6_0**	Orientation (phase) of the order-6, degree-0 Zernike moment of the Lamin B1 intensity distribution within the nucleus.
MeasureObjectIntensityDistribution	**ZernikePhase_OrigGreen_6_2**	Orientation (phase) of the order-6, degree-2 Zernike moment of the Lamin B1 intensity distribution within the nucleus.
MeasureObjectIntensityDistribution	**ZernikePhase_OrigGreen_6_4**	Orientation (phase) of the order-6, degree-4 Zernike moment of the Lamin B1 intensity distribution within the nucleus.
MeasureObjectIntensityDistribution	**ZernikePhase_OrigGreen_6_6**	Orientation (phase) of the order-6, degree-6 Zernike moment of the Lamin B1 intensity distribution within the nucleus.
MeasureObjectIntensityDistribution	**ZernikePhase_OrigGreen_7_1**	Orientation (phase) of the order-7, degree-1 Zernike moment of the Lamin B1 intensity distribution within the nucleus.
MeasureObjectIntensityDistribution	**ZernikePhase_OrigGreen_7_3**	Orientation (phase) of the order-7, degree-3 Zernike moment of the Lamin B1 intensity distribution within the nucleus.
MeasureObjectIntensityDistribution	**ZernikePhase_OrigGreen_7_5**	Orientation (phase) of the order-7, degree-5 Zernike moment of the Lamin B1 intensity distribution within the nucleus.
MeasureObjectIntensityDistribution	**ZernikePhase_OrigGreen_7_7**	Orientation (phase) of the order-7, degree-7 Zernike moment of the Lamin B1 intensity distribution within the nucleus.
MeasureObjectIntensityDistribution	**ZernikePhase_OrigGreen_8_0**	Orientation (phase) of the order-8, degree-0 Zernike moment of the Lamin B1 intensity distribution within the nucleus.
MeasureObjectIntensityDistribution	**ZernikePhase_OrigGreen_8_2**	Orientation (phase) of the order-8, degree-2 Zernike moment of the Lamin B1 intensity distribution within the nucleus.
MeasureObjectIntensityDistribution	**ZernikePhase_OrigGreen_8_4**	Orientation (phase) of the order-8, degree-4 Zernike moment of the Lamin B1 intensity distribution within the nucleus.
MeasureObjectIntensityDistribution	**ZernikePhase_OrigGreen_8_6**	Orientation (phase) of the order-8, degree-6 Zernike moment of the Lamin B1 intensity distribution within the nucleus.
MeasureObjectIntensityDistribution	**ZernikePhase_OrigGreen_8_8**	Orientation (phase) of the order-8, degree-8 Zernike moment of the Lamin B1 intensity distribution within the nucleus.
MeasureObjectIntensityDistribution	**ZernikePhase_OrigGreen_9_1**	Orientation (phase) of the order-9, degree-1 Zernike moment of the Lamin B1 intensity distribution within the nucleus.
MeasureObjectIntensityDistribution	**ZernikePhase_OrigGreen_9_3**	Orientation (phase) of the order-9, degree-3 Zernike moment of the Lamin B1 intensity distribution within the nucleus.
MeasureObjectIntensityDistribution	**ZernikePhase_OrigGreen_9_5**	Orientation (phase) of the order-9, degree-5 Zernike moment of the Lamin B1 intensity distribution within the nucleus.
MeasureObjectIntensityDistribution	**ZernikePhase_OrigGreen_9_7**	Orientation (phase) of the order-9, degree-7 Zernike moment of the Lamin B1 intensity distribution within the nucleus.
MeasureObjectIntensityDistribution	**ZernikePhase_OrigGreen_9_9**	Orientation (phase) of the order-9, degree-9 Zernike moment of the Lamin B1 intensity distribution within the nucleus.
